# Enhanced Honey Bee Colony Strength and Economic Returns from Fall and Winter Feeding with a Complete Pollen-Replacing Feed

**DOI:** 10.3390/insects17030243

**Published:** 2026-02-26

**Authors:** Kelly Kulhanek, Jan Bogaert, Anne Marie Fauvel, Brandon Hopkins, Thierry Bogaert

**Affiliations:** 1 Department of Entomology, Washington State University, Pullman, WA 99164-6382, USA; 2 APIX Biosciences NV, Blauwhuisstraat 11, 8750 Wingene, Belgium; 3 Bee Informed Partnership, College Park, MD 20742-0001, USA

**Keywords:** honey bee, nutrition, pollen-replacing feed, pollination management, feeding practices

## Abstract

Poor nutrition is a known contributing factor to ongoing high rates of honey bee colony mortality. Beekeepers invest significant resources to provide supplemental feeds to their colonies, but currently available diets are nutritionally incomplete. To test whether commercially managed colonies fed a manufactured, nutritionally complete Pollen-Replacing Feed (PRF-1) would exhibit improved colony health outcomes compared to beekeeper-selected Commercial Standard Feeds, we tracked colony health metrics from fall through almond pollination and the subsequent spring in a large-scale, multi-year field trial. By January (in almonds), PRF-1-fed colonies had 1.19 more frames of bees (*p* < 0.001) and 18.7% more colonies meeting the 8-frame minimum size requirement for high-revenue pollination contracts. After almond pollination (March), PRF-1-fed colonies exhibited a 13.8% increase in survival (*p* = 0.002), 2.57 more frames of bees (*p* = 0.006), and 0.79 more frames of brood (*p* = 0.003). PRF-1-fed colonies also exhibited superior spring build-up, adding 1.22 more frames of bees between January and March (*p* = 0.03). Economically, a hypothetical 100-colony operation fed PRF-1 garnered an additional $12,065.81 in gross revenue in the first year. Nutritional benefits are projected to compound, leading to exponentially increased revenue over subsequent years. Persistent improvements in colony health demonstrate that providing a nutritionally complete pollen-replacing feed in fall and winter has a long-lasting, positive impact on colony health and commercial viability.

## Simple Summary

Commercial honey bee colonies continue to face high rates of annual mortality, often exacerbated by poor nutrition, particularly during the transition from fall into the critical spring pollination season. Current commercial protein supplements (i.e., pollen substitutes), while widely used, are nutritionally incomplete. We hypothesized that feeding a nutritionally complete Pollen-Replacing Feed (PRF-1) over fall and winter would generate healthier, higher-quality winter bees compared to colonies fed the beekeeper’s selected commercially available feed. We conducted a large-scale, multi-year field trial across commercial operations, tracking colony health metrics starting in the fall through almond pollination and assessing subsequent spring build-up and economic outcomes. Colonies fed PRF-1 showed significantly improved health outcomes compared to the selected commercial diet group. Nutritionally complete supplemental feeding resulted in increased adult and immature bee populations and colony survival, which translates into economic advantages through increased pollination fees and colony growth (splitting) potential. The availability of a nutritionally complete pollen-replacing feed allows opportunities for improved management practices instrumental for commercial honey bee health and economic viability.

## 1. Introduction

European honey bees (*Apis mellifera*, L.) are critical for the pollination services of many fruit, nut, and specialty crops [1,2]. Honey bee colonies continue to experience high rates of annual mortality across the globe [3]. In the winter of 2024–2025, more colonies were lost in the U.S. than in any previously recorded year [4]. Research has identified several interacting causes of weakened colony health and decreased survival, including the parasitic mite *Varroa destructor*, pathogens, pesticides, and poor nutrition [5,6,7]. Poor nutrition weakens colonies and can also exacerbate negative effects from other stressors, including parasites, pathogens, and pesticide exposure [8,9,10].

A primary source of income for commercial beekeepers, particularly in the U.S., is the provision of crop pollination services [11]. To provide these services, beekeepers move their colonies into crop areas at high densities that are often nutritionally poor landscapes consisting of monocultures with few sources of natural pollen or nectar [12]. Participation in these pollination events can leave colonies nutritionally depleted and stressed, possibly inhibiting their potential for participation in future pollination events or honey production [13,14]. Beekeepers attempt to overcome the effects of poor nutritional landscapes with supplemental feeding of carbohydrates (sugar syrups) and protein (protein or pollen substitute or supplement) [15]. Beekeepers have been feeding artificial protein supplements to their colonies for decades, yet honey bees are the only major U.S. livestock without a manufactured, nutritionally complete, commercially available feed [16,17].

Honey bees acquire protein and essential nutrients from pollen, and beekeepers feed artificial pollen substitutes when natural pollen is scarce, notably in the fall when floral resources are minimal [9,18]. Feeding of pollen substitute in fall and winter has been increasingly common for U.S. beekeepers participating in early pollination events, such as almond pollination [19]. Currently available pollen substitutes can improve colony strength and size over winter, compared to not feeding [20,21,22]. However, these artificial feeds lack essential nutrients, including amino acids, are not digested as completely as natural pollen, and are associated with elevated pathogen levels compared to natural pollen [19]. Supplemental feed is the largest financial expense in commercial beekeeping operations each year [23,24], indicating that beekeepers deem it essential and expect a meaningful return on this investment.

At the end of the pollination season, colonies return weakened from serial pollination services, inadequate nutrition, pesticide exposure, transport stresses, and holding yard density stress [7,13]. Additionally, the end of summer and fall are periods with few flowering crops and limited flowering of natural vegetation. All these factors impact the number and quality of winter bees produced [25]. Winter bees are a specific long-lived caste of honey bees with a metabolism designed to survive to the next spring, characterized by their enlarged fat bodies [26]. In spring, these winter bees forage and nurse a new generation of brood. The number of winter bees, their lipid reserves, and their overall fitness are important to survive the winter, to replenish depleted food reserves, and to build a new generation of nurse bees in the spring when pollen and nectar are available again.

APIX Pollen (henceforth referred to as Pollen-Replacing Feed 1 [PRF-1]) is a nutritionally complete feed formulated to fulfill all nutrient requirements of honey bees. PRF-1 comprises a manufactured mixture of ingredients that are allowed in livestock feed applications in the E.U. and U.S. In prior work on different iterations of Pollen-Replacing Feed, honey bee colonies placed in tents from May to September and fed only a Pollen-Replacing Feed and sugar syrup maintained brood production for more than five consecutive brood cycles and more than two replacements of all the bees in the colonies [27]. Control colonies fed commercial standard diets showed reduced brood production and stopped brood production entirely after two brood cycles, hence becoming non-viable in the 105–120-day experiment [27]. A nutritionally complete manufactured diet (a Pollen-Replacing Feed) requires the presence of a multitude of ingredients in specific concentrations and balanced ratios (lipids, amino acids, sterols, vitamins, minerals, antioxidants, etc.). The specific recipe is proprietary company information and based on Diets A and D in Bogaert et al. 2025 [27].

Here, we aimed to test the efficacy of the first nutritionally complete artificial feed for honey bees compared to the existing best feeding practices of experienced and successful commercial beekeepers. We hypothesized that honey bee colonies fed a nutritionally complete pollen-replacing feed (PRF-1) over fall and winter would generate healthier winter bees compared to colonies fed commonly available and widely used commercial standard protein supplements. We conducted a large-scale field trial to assess colony strength in almond pollination and after almond pollination to evaluate whether effects from fall and winter nutritional inputs persist into the following spring and next beekeeping season. Finally, we assessed colony growth from January to March (“spring build-up”) on a set of colonies fed either PRF-1 or a beekeeper-selected Commercial Standard Feed over the previous fall and winter to test whether higher quality winter bees increase colony population size more quickly, regardless of their starting population size. This is the largest-scale and longest-term trial of honey bee protein supplements to date. It is also the only trial conducted entirely in commercial beekeeping conditions, with the beekeepers themselves performing colony management and feeding. This study tests the effects of the first nutritionally complete feed for honey bees under real-world conditions and across variable beekeeping operations and management practices, landscapes, and colony health stressors.

## 2. Materials and Methods

### 2.1. Beekeeping Operations, Colony Selection, and Feeding

Colonies were selected for monitoring from five commercial beekeeping operations in California and Idaho beginning in the fall (late August or early September) of 2022 and 2023. Beekeepers were asked to provide queenright colonies at the end of August. These professional beekeepers each manage more than 2000 colonies and already practice supplemental feeding with protein supplements and sugar syrup in the fall, using well-known commercially available protein supplements. The colonies of each beekeeper were divided into two treatment groups. One treatment group received an artificial nutritionally complete feed referred to as Pollen-Replacing Feed 1 (PRF-1). The other treatment group received the protein supplement already used in the beekeepers’ operations (Commercial Standard Feed). The protein supplements used by the participating beekeepers were from diverse manufacturers and are widely used in the U.S. Each beekeeper was free to select their chosen Commercial Standard Feed, each of which was a commercially available protein supplement specifically produced for honey bee feeding. In this way, the effect of PRF-1 is compared to the standard commercial practice of these beekeepers.

One beekeeper was based in Southern California (San Diego area) and provided supplemental feeding through fall and winter, as bees remain active in the warmer climate, but pollen is scarce. One beekeeper was based in Idaho and provided supplemental feeding in the fall until colonies were placed in an indoor storage building from early November through the end of January. Colonies were shipped to almond pollination upon removal from indoor storage and were fed immediately upon arrival in California. The other beekeepers were based in Northern California (north of Sacramento) and provided supplemental feeding in the fall period until the weather gets too cold to open the colonies (early November) and were then fed again upon arrival to almond pollination in January. The management practices and locations of each operation were unique, but within each operation, all colonies, regardless of treatment group, were treated the same in every way except the experimental diet they received. This allows for assessment of PRF-1’s effects across a diverse set of commercial beekeeping operations.

Colonies of the Commercial Standard Feed treatment group followed each beekeeper’s normal protein supplement feeding practice: typically 1–2 lbs (0.45–0.91 kg) every two weeks for a colony with 6–15 frames of bees. Beekeepers were instructed to feed colonies of the PRF-1 treatment group with 1.5 lbs (0.68 kg) of PRF-1 for a colony with 6–15 frames of bees, following the feeding regimen of PRF-1. In all other aspects, colonies of both treatment groups were treated the same, according to the beekeepers’ usual commercial practices (*Varroa* treatment, sugar syrup feeding, etc.). Feeding according to assigned treatment groups began in the fall at the initial colony inspection and selection and concluded in late January or early February prior to almond pollination.

Each operation was asked to provide 160 colonies (80 per treatment group). Colonies starting with fewer than 6 frames of bees were excluded from the study as these were deemed unlikely to survive fall and winter. Colonies with more than 15 frames of bees were excluded, as the recommended dose of both PRF-1 and the Commercial Standard Feed was targeted at colonies with up to 15 frames of bees. After excluding colonies outside the 6–15 frames of bees range, each treatment group had the following number of colonies at the start of monitoring in fall: “in almonds” PRF-1 *n* = 382, Commercial Standard *n* = 382; “after almonds” PRF-1 *n* = 141, Commercial Standard *n* = 147; “Spring Build-Up” PRF-1 *n* = 93, Commercial Standard *n* = 92. There was no difference in average colony size between treatment groups at the start of monitoring in the fall for any cohort.

Beekeepers were asked to keep colonies together in one yard if possible, or if in multiple yards, have colonies of each treatment group equally present in each yard. Colonies were moved according to the beekeepers’ normal operations (i.e., to almond pollination) throughout the course of the study.

### 2.2. Colony Inspection

Depending on the availability of each beekeeper and their colonies for the performance of assessments, some operations were assessed at three time points in fall, January, and March (*n* = 2 operations in 2022; *n* = 2 operations in 2023), some were assessed only in fall and January (*n* = 2 operations in 2022; *n* = 1 operation in 2023), and one was assessed only in fall and March (*n* = 1 operation in 2022). We divided operations into two cohorts: those with a fall and January assessment (*n* = 4 operations in 2022; *n* = 3 operations in 2023) and those with a fall and March assessment (*n* = 3 operations in 2022; *n* = 1 operation in 2023). The first assessment conducted on colonies in the fall coincided with the onset of feeding the feeds assigned to each treatment group. Colonies assessed in January are referred to as the “in almonds” group, and colonies assessed in March are referred to as the “after almonds” group. The following colony health measurements were taken at each assessment according to standard methods: queen status, frames of bees (subjective mode), and frames of capped brood (subjective mode) [28]. In the first study year (2022–2023), a random subset of 240 colonies from three operations (40 colonies from each diet group in each operation) was sampled for assessment of Varroa load in fall and January according to standard methods [28]. A random subset of these 240 colonies (*n* = 120; 20 colonies from each diet group in each operation) had additional samples taken for pathogen prevalence and quantification according to standard methods [28]. Varroa infestation and viral infection did not differ between diet groups at any time point, so these results are included in Supplementary Material (Figures S1–S3). Colonies where queen issues were recorded during assessments (queenless, drone-laying, laying worker) were excluded from analyses. Colonies that were found dead upon scheduled inspection were recorded as such. Colonies missing from the yard were also recorded as dead according to the beekeeper’s specification that any missing colonies would have either been found dead and removed from the yard or were so weak they were considered effectively dead and were combined into other colonies.

### 2.3. Spring Build-Up Treatment Group Assignments

To assess “spring build-up,” or colony population growth from the time interval between January and March, two new treatment groups were formed in one Southern California commercial beekeeping operation in January 2024. One treatment group consisted of colonies that had been fed PRF-1 (*n* = 93), and the other of colonies fed according to a Commercial Standard Feed (*n* = 92) as described above. Colonies were selected based on population size in frames of bees so that colonies were between 6 and 15 frames of bees, and each treatment group had an average population size of approximately 10 frames of bees in January. Colonies with queen issues or obvious signs of disease were excluded. These colonies were assessed for frames of bees and queen status in January (in almonds) and March (in Southern California) according to standard methods [28].

### 2.4. Analyses

After initial inspection in the fall, colony health outcomes were assessed at three different time points: “in almonds,” “after almonds,” and “spring build-up.” Fall inspections were performed where each beekeeping operation was based: Idaho, Northern, and Southern California. “In almonds” colony health outcomes were assessed in January in almond orchards and included overall colony size (frames of bees) and the percent of colonies with at least eight frames of bees. Dead colonies were counted in frames of bees analyses as having 0 frames of bees and in percent of colonies with at least eight frames of bees analyses as not having 8 frames of bees. Colonies with more frames of bees can earn more revenue in almond pollination fees and may also be used to donate frames to other hives to make more colonies to participate in almond pollination. The most frequently reported average colony size reported in almond pollination contracts is eight frames of bees. The percent of colonies with at least eight frames of bees in January was calculated according to Equation (1):

(1) $$\%\;colonies\;with\geq 8\;frames\;of\;bees\;in\;January=\left(\frac{\#\;colonies\;with\geq 8\;frames\;of\;bees\;in\;January}{\#\;colonies\;alive\;in\;fall}\right)\times 100$$

“After almonds” colony health outcomes were assessed in March, where each operation was based (Idaho, Northern, or Southern California) and included frames of bees, frames of capped brood, and the percent of colonies alive. Dead colonies were counted in frames of bees and frames of capped brood analyses as having 0 frames of bees and brood. These metrics are indicative of the overall strength of the colonies and operation, as well as how many splits, nucleus colonies, or packages could be produced to either participate in further pollination events and/or honey production or be sold. The percent of colonies alive in March was calculated according to Equation (2):

(2) $$\%\;colonies\;alive\;in\;March\;=\;\left(\frac{\#\;colonies\;alive\;in\;March\;}{\#\;colonies\;alive\;in\;fall}\right)\times 100$$

All analyses were conducted in R (version 4.5.1 “Great Square Root”). Summary statistics are reported as mean ± one standard error of the mean or as a percentage with a 95% confidence interval when appropriate. Linear mixed-effects models were used to check for significant effects of variables with repeated measures (lmer function from the ‘lme4’ library) [29]. Non-significant model terms were eliminated in a stepwise fashion using analyses of deviance (ANOVA function from the “stat” package). Binomial mixed-effects models were used in the same fashion for colonies with at least eight frames of bees in January and colonies alive in March, with “colony has eight or more than eight frames” or “colony has fewer than eight frames” and “dead or missing” and “not dead or missing” as the possible outcomes for each model (glmer function from “lme4” library) [29]. We assessed each metric for significant differences between diet groups and also checked for differences in the effects of diet across operations by assessing the interaction between diet group and operation. P-values for comparisons are included in the text, and p-values smaller than 0.001 are reported as *p* < 0.001. Significant differences are represented on figures with asterisks according to the following significance levels: * *p* < 0.05, ** *p* < 0.01, and *** *p* < 0.001.

### 2.5. Economic Analysis Methods

For the economic analysis, we began with a hypothetical operation of 100 colonies that were fed either PRF-1 or a Commercial Standard Feed beginning in the fall. We calculated the number of colonies expected to have at least eight frames of bees in January (almond pollination) based on the percentage of colonies with at least eight frames of bees in the “in almonds” cohort of the field trial. Eight frames is the most frequently reported average frame count specified by almond growers in pollination contracts [30]. Almond pollination contracts paid $181 (USD) per hive on average in 2024 [31].

To assess economic differences between treatment groups in March, we considered the number of five-frame colonies that could be produced in each treatment group and assessed the potential gross revenue generated by these frames of bees in different conditions throughout the following year. We considered a hypothetical situation where all frames of bees in an operation would be used to generate five-frame nucleus colonies or “splits,” that can continue to subsequent pollination events (i.e., apple pollination) and honey production. The number of five-frame colonies produced was calculated by multiplying the mean colony size (frames of bees) in March (from the “after almonds” cohort) by the number of colonies alive in the operation the previous fall. Winter colony mortality is considered in this calculation as the mean frames of bees, which includes dead colonies as having 0 frames of bees. Apple pollination contracts paid $58.10 per hive on average in 2024 [31]. The average colony produced 51.7 lbs of honey, and honey generated $2.69 of revenue per lb in 2024 for a total honey production revenue of $1439.07 per hive on average [24].

To assess how colony health differences and associated revenue would compound over multiple years, we performed a hypothetical analysis assuming that colony health differences remain constant over a five-year period. This analysis assumed the following metrics remain constant over a five-year period: the percentage of colonies with at least eight frames of bees in January, the mean frames of bees per hive in March, and the percentage of colonies lost over the summer. The percent of colonies lost over the summer used in this analysis was 23.6%, the 14-year average (2010–2023) for summer loss in commercial operations according to national annual surveys [4]. For each subsequent year, we re-applied these percentages to calculate the operation size that would participate in almond pollination, apple pollination, and honey production, as well as associated revenue. For this exercise, we assumed revenue from these activities remains the same over the five-year period. For example, a PRF-1-fed hive in Year 2 would begin the year in fall after honey production with 148.52 colonies (194.4 colonies produced in the previous March × 0.764). We assumed 67.89% of these colonies would have at least eight frames of bees in January for a total of 100.83 colonies participating in almond pollination. We then multiplied the number of colonies in fall (148.52) by the mean colony size in March (9.72 frames of bees) to get the total number of frames of bees available in March (1443.63) and divided by five to get the number of five-frame colonies produced (288.73). These colonies would then go on to participate in apple pollination and honey production, and the calculations are repeated for a total of five years.

## 3. Results

### 3.1. Colony Strength in Almonds

**Figure 1:**
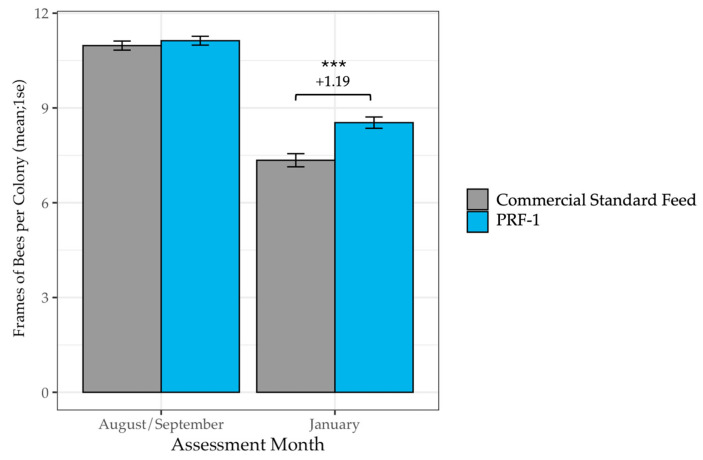
Mean frames of bees per colony in PRF-1 and Commercial Standard-fed colonies at the start of the study in the fall and in January. In January, colonies fed PRF-1 had, on average, 1.19 more frames of bees than colonies fed a Commercial Standard Feed (*F*_1_ = 20.24, *p* < 0.001). The level of the significant difference is denoted by *** (*p* < 0.001).

Feeding a more complete nutritional supplement provided a significant advantage leading to almond pollination. A total of 385 PRF-1-fed colonies and 382 Commercial Standard-fed colonies were included in the analysis. While there was no initial significant difference in colony size between treatment groups in the fall (*F*_1_ = 0.01, *p* = 0.90), by January, colonies fed PRF-1 had on average 1.19 more frames of bees than colonies fed a Commercial Standard (Table 1, Figure 1, *F*_1_ = 20.24, *p* < 0.001). PRF-1-fed colonies had 16% more frames of bees on average than Commercial Standard colonies in January. Importantly, while colony size varied naturally across different beekeeping operations (*F*_3_ = 5.05, *p* < 0.001), the positive effect of PRF-1 was consistent across all operations (operation × treatment group interaction: *F*_3_ = 0.75, *p* = 0.57).

**Figure 2:**
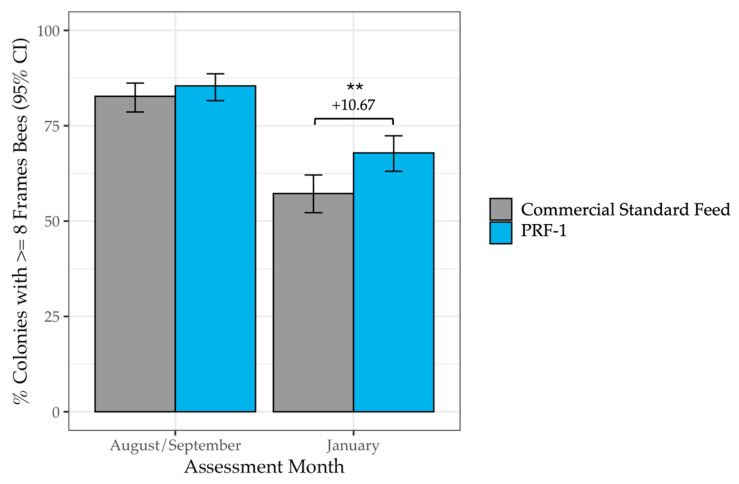
Percent of colonies with at least 8 frames of bees in PRF-1 and Commercial Standard-fed colonies at the start of the study in fall and in January. In January, a total of 67.89% of PRF-1-fed colonies had at least eight frames of bees in January, compared to a total of 57.22% of Commercial Standard Fed colonies (*F*_1_ = 9.30, *p* = 0.002). The level of the significant difference is denoted by ** (*p* < 0.01).

The size advantage of feeding the complete dietary supplement translated into a higher proportion of rentable colonies (at least eight frames of bees). In January, 67.89% of PRF-1-fed colonies had at least eight frames of bees, compared to only 57.22% of Commercial Standard-fed colonies (Table 2, Figure 2, *F*_1_ = 9.30, *p* = 0.002). This represents a 10.67 percentage point increase in premium pollination units. Although the percentage of large colonies varied by year and operation, the benefit of PRF-1 was consistent across all contexts (feed × year: *p* = 0.67; feed × operation: *p* = 0.29). There was no significant difference in the percentage of colonies with at least eight frames of bees at the start in fall (*F*_1_ = 1.07, *p* = 0.30).

### 3.2. Colony Strength After Almonds

**Figure 3:**
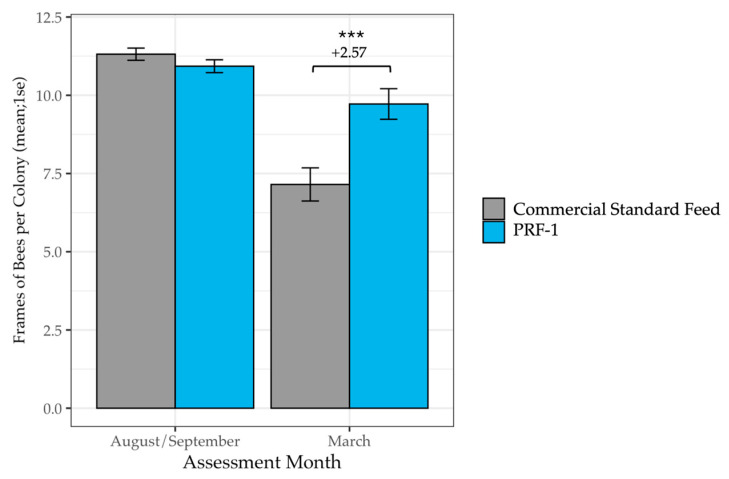
Mean frames of bees per colony in PRF-1 and Commercial Standard-fed colonies at the start of the study in fall and in March. In March, colonies fed PRF-1 had, on average, 2.57 more frames of bees than colonies fed a Commercial Standard Feed (*F*_1_ = 7.81, *p* = 0.006). The level of the significant difference is denoted by *** (*p* < 0.001).

**Figure 4:**
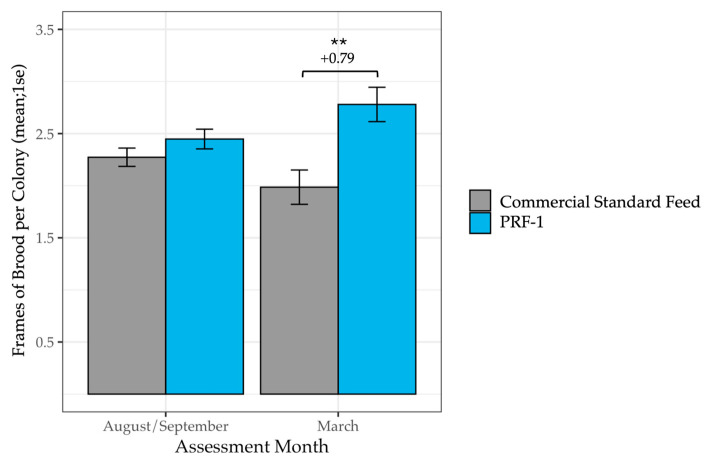
Mean frames of brood per colony in PRF-1 and Commercial Standard-fed colonies at the start of the study in fall and in March. In March, colonies fed PRF-1 had, on average, 0.79 more frames of brood than colonies fed a Commercial Standard Feed (*F*_1_ = 9.12, *p* = 0.003). The level of the significant difference is denoted by ** (*p* < 0.01).

The benefits of the feeding persisted through the almond bloom. In the March analysis (*n* = 141 PRF-1, *n* = 147 Commercial), PRF-1 colonies maintained significantly larger populations and brood areas. Despite starting with equal colony sizes in the fall (*F*_1_ = 1.87, *p* = 0.17), by March, PRF-1 colonies contained an average of 2.57 more frames of bees (*F*_1_ = 7.81, *p* = 0.006; Table 3, Figure 3) and 0.79 more frames of brood (*F*_1_ = 9.12, *p* = 0.003; Table 4, Figure 4) than the Commercial Standard group. These differences represent a 36% increase in population and a 40% increase in brood production. As with the January data, these effects were consistent across different beekeeping operations (operation × treatment group interaction: *p >* 0.05).

**Figure 5:**
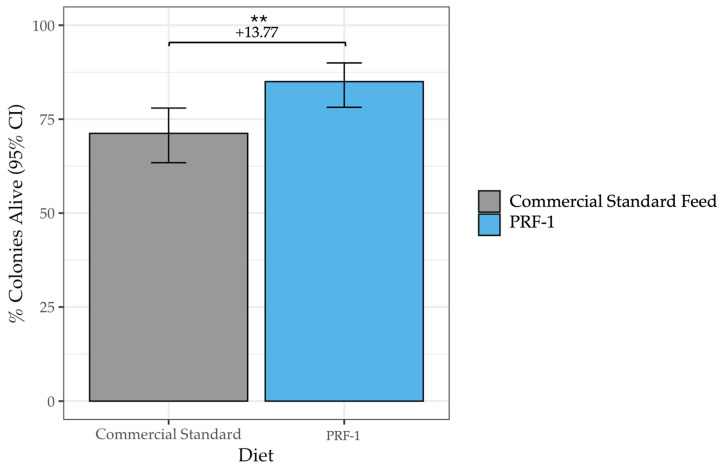
Percent of PRF-1 and Commercial Standard-fed colonies alive in March. A total of 85.00% of PRF-1-fed colonies were alive in March, compared to 71.23% of Commercial Standard Fed colonies (*F*_1_ = 9.90, *p* = 0.002). The level of the significant difference is denoted by ** (*p* < 0.01).

Most notably, PRF-1 significantly improved overwinter survival. A total of 85.0% of PRF-1 colonies were alive in March, compared to only 71.23% of Commercial Standard-fed colonies (*F*_1_ = 9.90, *p* = 0.002; Table 5, Figure 5). This constitutes a 13.77 percentage increase in survivorship. Survival rates did not differ significantly by year or beekeeping operation, indicating that the feeding was the primary driver of reduced mortality.

### 3.3. Spring Build Up

**Figure 6:**
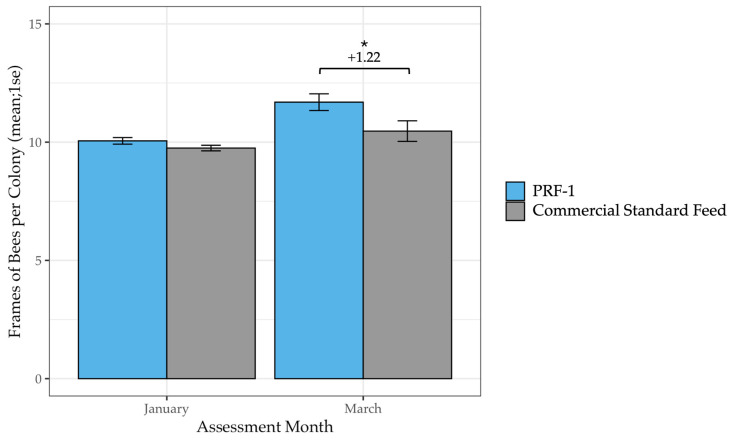
Mean frames of bees per colony in PRF-1 and Commercial Standard-fed colonies in January and March, when equalized to a size of approximately 10 frames of bees in January. By March, colonies fed PRF-1 had on average 1.22 more frames of bees than colonies fed a Commercial Standard Feed (*F*_1_ = 4.70, *p* = 0.03). The level of the significant difference is denoted by * (*p* < 0.05).

To isolate the effect of the diet on spring growth rates (independent of winter survival), we analyzed a subset of colonies where colony sizes were equalized in January (*n* = 93 PRF-1, *n* = 92 Commercial; baseline difference *F*_1_ = 2.76, *p* = 0.10). By March, the PRF-1 colonies had clearly outperformed the Commercial Standard group, averaging 1.22 more frames of bees (F_1_ = 4.70, *p* = 0.03; Table 6, Figure 6). This represents a 12% increase in colony size during the critical spring build-up period, demonstrating that PRF-1 supports faster population expansion even when starting from an equal baseline.

### 3.4. Economic Analysis Results

#### 3.4.1. Projected Revenue for Year 1

The biological advantages of PRF-1 translated directly into economic gains, driven by higher pollination grades in January and increased biological assets in March. First, regarding almond pollination revenue: based on the January assessment, where 67.89% of PRF-1 colonies met the 8-frame grade versus 57.22% of Commercial Standard colonies (*p* = 0.002), a theoretical operation of 100 colonies would realize significantly higher rental fees. Specifically, the PRF-1 diet yields an additional 10.67 grade-eligible colonies per 100 hives. At a rental rate of $181 per colony, this generates an additional $1931.27 in gross revenue for the almond pollination season (Table 7).

Second, regarding post-pollination value: The economic gap widens significantly in March when accounting for survival and colony size. In a 100-colony model, the PRF-1 group finishes the season with a larger average population per colony (9.72 frames vs. 7.15 frames). This measurement of frames of bees includes dead colonies as having 0 frames of bees. The difference in survival and colony size creates a massive difference in total biological inventory: 972.00 total frames for PRF-1 versus 715.00 for the standard feed. If broken down into 5-frame nucleus colonies (nucs) for sale or expansion, the PRF-1 operation produces 194.40 nucs compared to only 143.00 for the standard feed—a gain of 51.40 additional nucs.

When combining almond pollination fees, subsequent apple pollination, and honey production/splits, the PRF-1 regimen generates an estimated $12,065.81 in additional revenue per 100 colonies. This equates to an added value of $120.66 per colony.

#### 3.4.2. Projected Gross Revenue for Five Years

**Figure 7:**
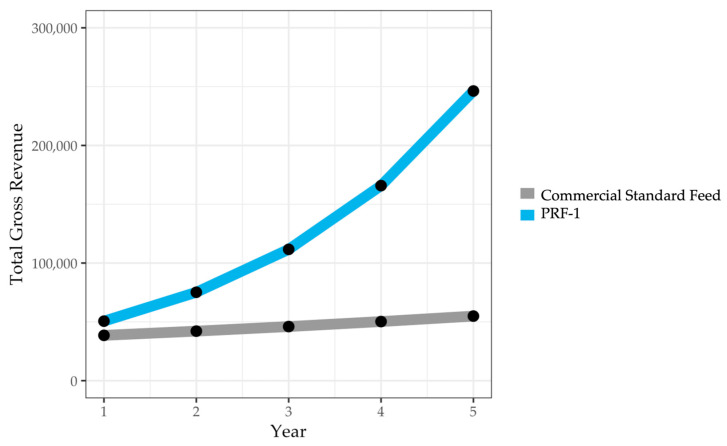
Projected gross revenue from almond and apple pollination and honey production for five years when feeding with either PRF-1 or a Commercial Standard Feed in fall and winter.

Assuming hive health and economic metrics used to calculate gross revenue in Year 1 remained constant for five years, and assuming 23.6% summer colony loss (or 76.4% summer survival), the PRF-1-fed operation would begin Year 2 in the fall after honey production with 148.52 colonies (194.4 colonies produced in the previous March × 0.764). For almond pollination in January, 67.89% of these colonies would have at least eight frames of bees for a total of 100.83 colonies (Table 8). With a fall operation size of 148.52 colonies and a mean colony size of 9.72 frames of bees, overwintered colonies represent a total of 1443.63 frames of bees, which would produce 288.73 five-frame nucleus colonies in March. These colonies then go on to participate in apple pollination and honey production, and the calculations are repeated for a total of five years. By Year 5, PRF-1-fed colonies would generate a gross revenue of $246,299.16, compared to $54,924.48 in Commercial Standard-fed colonies (Figure 7).

## 4. Discussion

We found that colonies fed PRF-1 exhibited increased colony strength in almond pollination compared to colonies fed Commercial Standard Feeds. A nutritionally complete fall and winter feed contributed to larger colony sizes in almonds and more colonies that can participate in almond pollination. Increased colony strength represents improved income potential for commercial beekeepers in almonds, as larger colonies garner a higher price [30,32].

The effect of fall and winter feeding practices persisted after almond pollination into spring. Colonies fed PRF-1 exhibited increased colony survival and had more brood and adult bees than colonies fed according to commercial standard beekeeping practices in March. Almond pollen is of very high nutritional quality to honey bees, so one may expect that after almond pollination, any effect of fall or winter diet would be superseded by the nutritional inputs of an influx of almond pollen [33]. PRF-1-fed colonies exhibited increased colony strength and survival across all beekeeping operations, indicating that although operations differed in management practices and locations, the effects of the nutritionally complete diet were persistent. This persistent improvement in colony strength in PRF-1-fed colonies is demonstrative of the lasting effects of nutritional inputs.

An increase in the spring quantity of living colonies, adult bees, and brood represents increased income potential for commercial beekeepers either from using the surplus to create packages and nucleus colonies to sell or to create more colonies via “splitting” to garner additional income in subsequent pollination events and/or honey production. Fall and winter nutrition likely has impacts on colony strength and income potential for at least the subsequent beekeeping season. We further demonstrated with the economic analysis that these effects could accumulate over subsequent years, where each year, income potential is increased as a result of improved winter survival and spring colony strength.

The persistent effects of fall and winter diet were also evident during the “spring build-up” period. Colonies that had been fed PRF-1 the previous fall and winter exhibited increased population growth compared to colonies fed Commercial Standard Feeds, even when starting from equal colony population sizes in January. This result indicates that colonies fed a nutritionally complete pollen-replacing feed likely produced higher-quality winter bees, which were able to rear more brood in the spring. The quality of winter bees appears to be a more important indicator of spring population growth than the quantity of winter bees.

The colonies included in this study, particularly colonies fed PRF-1, exhibited much lower rates of mortality than the national average winter loss rate among commercial beekeeping operations (15.0% in PRF-1-fed colonies and 28.9% in Commercial Standard-fed colonies compared to 40.7% national average in 2024–2025 [4]). Colonies included in the study were selected to have at least 6 frames of bees and no queen issues or visible signs of disease at the start of the study, which may contribute to their reduced mortality compared to the national average. Even with low relative colony loss rates, PRF-1-fed hives still exhibited increased colony survival, indicating that the diet could improve outcomes compared to the highest quality commercial standard beekeeping practices.

This study was conducted truly in situ in commercial beekeeping conditions and is the largest-scale and longest-term study of nutritional inputs to commercial honey bee colonies. The beekeepers delivered both feeds to the colonies, and experimental colonies were managed within commercial beekeeping operations exactly as all the other colonies were managed. This demonstrates that providing complete nutritional supplementation improves colony health outcomes in real-world conditions, and results can be interpreted by beekeepers as directly applicable to their operations. There are clear effects of PRF-1 on colony performance across commercial operations despite the high variability in biotic and abiotic factors that affect colony health in a real-world setting.

Bees are the only livestock for which, until recently, no nutritionally complete pollen-replacing feed had been available, leading to a current beekeeping practice where nutritionally incomplete supplementary feeds are used routinely to bridge periods of pollen dearth or periods where only nutritionally inadequate, incomplete pollen is available for foraging. Bogaert et al. 2025 showed that continued feeding during a series of nutritionally stressful pollination events showed a step change in colony survival and growth [27]. This study builds on prior work by demonstrating improvements in colony strength, survival, and income potential resulting from feeding a nutritionally complete pollen-replacing feed in the winter months. Our findings have significant implications for the future of commercial honey bee colony management and crop pollination in the U.S. Providing more complete nutritional supplementation for colonies in the current landscape can significantly improve beekeeping operations, bolstering colony strength and survival. These impacts will benefit commercial beekeeping operations, as well as support the supply of colonies delivering essential pollination services for many crops.

## Figures and Tables

**Table 1:** Mean frames of bees per colony in PRF-1 and Commercial Standard-fed colonies at the start of the study in the fall and in January. In January, colonies fed PRF-1 had, on average, 1.19 more frames of bees than colonies fed a Commercial Standard Feed (*F*_1_ = 20.24, *p* < 0.001).

Diet	Assessment Month	n Colonies	Frames of Bees (Mean ± 1 S.E.)
PRF-1	August/September	385	11.13 ± 0.14
PRF-1	January	383	8.54 ± 0.18
Commercial Standard Feed	August/September	382	10.97 ± 0.14
Commercial Standard Feed	January	381	7.35 ± 0.21

**Table 2:** Percent of PRF-1-fed colonies and Commercial Standard-fed colonies with at least 8 frames of bees at the start of the study in fall and in January. A total of 10.67% more PRF-1-fed colonies had at least 8 frames of bees than Commercial Standard-fed colonies (*F*_1_ = 9.30, *p* = 0.002).

Diet	Assessment Month	n Colonies	% Colonies with ≥8 Frames of Bees [95% CI]
PRF-1	August/September	385	85.45 [81.58–88.63]
PRF-1	January	383	67.89 [63.05–72.26]
Commercial Standard Feed	August/September	382	82.72 [78.61–86.18]
Commercial Standard Feed	January	381	57.22 [52.20–62.10]

**Table 3:** Mean frames of bees per colony in PRF-1 and Commercial Standard-fed colonies at the start of the study in fall and in March. In March, colonies fed PRF-1 had, on average, 2.57 more frames of bees than colonies fed a Commercial Standard Feed (*F*_1_ = 7.81, *p* = 0.006).

Diet	Assessment Month	n Colonies	Frames of Bees (Mean ± 1 S.E.)
PRF-1	August/September	141	10.93 (±0.21)
PRF-1	March	140	9.72 (±0.49)
Commercial Standard Feed	August/September	147	11.31 (±0.19)
Commercial Standard Feed	March	146	7.15 (±0.53)

**Table 4:** Mean frames of brood per colony in PRF-1 and Commercial Standard-fed colonies at the start of the study in fall and in March. In March, colonies fed PRF-1 had, on average, 0.79 more frames of brood than colonies fed a Commercial Standard Feed (*F*_1_ = 9.12, *p* = 0.003).

Diet	Assessment Month	n Colonies	Frames of Brood (Mean ± 1 S.E.)
PRF-1	August/September	141	2.45 (±0.09)
PRF-1	March	140	2.78 (±0.16)
Commercial Standard Feed	August/September	147	2.27 (±0.09)
Commercial Standard Feed	March	146	1.99 (±0.16)

**Table 5:** Percent of PRF-1 and Commercial Standard-fed colonies alive in March. A total of 13.77% more PRF-1-fed hives were alive in March than Commercial Standard-fed colonies (*F*_1_ = 9.90, *p* = 0.002).

Diet	Assessment Month	n Colonies	% Colonies Alive [95% CI]
PRF-1	March	140	85.00 [78.16–89.97]
Commercial Standard Feed	March	146	71.23 [63.42–77.96]

**Table 6:** Mean frames of bees per colony in PRF-1 and Commercial Standard-fed colonies in January and March, when equalized to a size of approximately 10 frames of bees in January. By March, colonies fed PRF-1 had on average 1.22 more frames of bees than colonies fed a Commercial Standard Feed (*F*_1_ = 4.70, *p* = 0.03).

Diet	Assessment Month	n Colonies	Frames of Brood (Mean ± 1 S.E.)
PRF-1	January	93	10.05 (±0.14)
PRF-1	March	93	11.69 (±0.35)
Commercial Standard Feed	January	92	9.75 (±0.12)
Commercial Standard Feed	March	92	10.47 (±0.44)

**Table 7:** Colony health metrics and associated projected gross revenue from almond and apple pollination and/or honey production in the first year after fall and winter feeding with either PRF-1 or a Commercial Standard Feed, starting with an operation size of 100 colonies in fall. The difference between treatment groups in each colony health and economic metric is highlighted in light blue. The Total Gross Revenue in Year 1 for each treatment group and the difference between them is highlighted in dark blue.

In Almonds (Operation Consists of Overwintered Colonies from a Starting Point of 100 Colonies in the Fall)	PRF-1	Commercial Standard Feed	Difference
**Colony Health Metric**			
# Colonies with ≥ 8 frames of bees in January (% Colonies with ≥ 8 frames of bees in January × 100)	67.89	57.22	10.67
**Economic Metric**			
Gross revenue in almond pollination fees (# Colonies with ≥ 8 frames of bees in January × $181/hive)	$12,288.09	$10,356.82	$1931.27
**After almonds** (Operation consists of 5-frame colonies generated from all frames of bees available in March)	**PRF-1**	**Commercial Standard Feed**	**Difference**
**Colony Health Metric**			
Mean frames of bees per colony in March (includes dead colonies as 0 frames of bees)	9.72	7.15	2.57
Total frames of bees in March (Mean frames of bees per colony in March × # of colonies alive in fall)	972.00	715.00	257.00
# 5-frame colonies produced (Total frames of bees/5)	194.40	143.00	51.40
**Economic Metric**			
Gross revenue in apple pollination fees (# 5-frame colonies produced × $58.1/hive)	$11,294.64	$8308.30	$2986.34
Gross revenue in honey production (# 5-frame colonies produced × $139.07/hive)	$27,035.21	$19,887.01	$7148.20
**TOTAL Gross Revenue in Year 1**	**$50,617.94**	**$38,552.13**	**$12,065.81**

**Table 8:** Colony health metrics and associated projected gross revenue from almond and apple pollination and honey production in the second through fifth years when feeding with either PRF-1 or a Commercial Standard Feed in fall and winter. “In-almonds” metrics are in white and “after-almonds” metrics are in light gray. Total Gross Revenue for each treatment group each year is highlighted in light blue and the difference in Total Gross Revenue between treatment groups each year is highlighted in dark blue.

Year	Colony Health and Economic Metrics	PRF-1	Commercial Standard Feed	Difference
**2**	# Hives in Almonds	100.83	62.51	38.32
	Gross Revenue in Almond Pollination Fees	$18,250.47	$11,315.03	$6935.43
	Total Frames of Bees in March	1443.63	781.15	662.48
	# 5-frame Hives Produced in March	288.73	156.23	132.50
	Gross Revenue in Apple Pollination Fees	$16,774.98	$9076.98	$7698.00
	Gross Revenue in Honey Production	$40,153.12	$21,726.96	$18,426.17
	TOTAL Gross Revenue	$75,178.57	$42,118.97	$33,059.60
**3**	# Hives in Almonds	149.76	68.30	81.46
	Gross Revenue in Almond Pollination Fees	$27,105.89	$12,361.90	$14,743.99
	Total Frames of Bees in March	2144.10	853.42	1290.68
	# 5-frame Hives Produced in March	428.82	170.68	258.14
	Gross Revenue in Apple Pollination Fees	$24,914.47	$9916.79	$14,997.68
	Gross Revenue in Honey Production	$59,636.06	$23,737.13	$35,898.93
	TOTAL Gross Revenue	$111,656.42	$46,015.82	$65,640.60
**4**	# Hives in Almonds	222.42	74.62	147.80
	Gross Revenue in Almond Pollination Fees	$40,258.10	$13,505.62	$26,752.47
	Total Frames of Bees in March	3184.46	932.38	2252.07
	# 5-frame Hives Produced in March	636.89	186.48	450.41
	Gross Revenue in Apple Pollination Fees	$37,003.37	$10,834.29	$26,169.08
	Gross Revenue in Honey Production	$88,572.43	$25,933.29	$62,639.14
	TOTAL Gross Revenue	$165,833.90	$50,273.20	$115,560.69
**5**	# Hives in Almonds	330.34	81.52	248.82
	Gross Revenue in Almond Pollination Fees	$59,791.97	$14,755.16	$45,036.81
	Total Frames of Bees in March	4729.60	1018.65	3710.96
	# 5-frame Hives Produced in March	945.92	203.73	742.19
	Gross Revenue in Apple Pollination Fees	$54,957.99	$11,836.68	$43,121.32
	Gross Revenue in Honey Production	$131,549.19	$28,332.64	$103,216.55
	TOTAL Gross Revenue	$246,299.16	$54,924.48	$191,374.68

## Data Availability

The data presented in this study are available on request from the corresponding author due to Intellectual Property Management.

## Supplementary Materials

The following supporting information can be downloaded at: https://www.mdpi.com/article/10.3390/insects17030243/s1, Figure S1: Mean Varroa loads (mites per 100 bees) per colony in PRF-1 (*n* = 120) and Commercial Standard-fed (*n* = 120) colonies at the start of the study in fall and in January. There was no difference in Varroa load between treatment groups (*F*_1_ = 0.1397 *p* = 0.71); Figure S2: Pathogen prevalence (proportion of samples with each pathogen detected) in PRF-1 (*n* = 60) and Commercial Standard-fed (*n* = 60) colonies at the start of the study in fall and in January. Pathogens assessed include: Acute Bee Paralysis Virus (ABPV), Chronic Bee Paralysis Virus (CBPV), Deformed Wing Viruses A and B (DWV-A, DWV-B), Israeli Acute Paralysis Virus (IAPV), Kashmir Bee Virus (KBV), Lake Sinai Virus (LSV), and *Nosema ceranae* (NC). There were no significant differences between treatment groups for any pathogen: ABPV *F*_1_ = 2.91, *p* = 0.09; CBPV *F*_1_ = 0.01, *p* = 0.94; DWV-A *F*_1_ = 0.15, *p* = 0.70; DWV-B *F*_1_ = 0.00, *p* = 1.00; IAPV *F*_1_ = 0.001, *p* = 0.97; KBV *F*_1_ = 0.00, *p* = 1.00; LSV *F*_1_ = 3.74, *p* = 0.06; NC *F*_1_ = 0.24, *p* = 0.62. Figure S3: Fold difference in quantified pathogen expression between PRF-1 (*n* = 60) and Commercial Standard-fed (*n* = 60) colonies as the start of the study in fall and in January. Pathogen expression is presented at the fold difference between the number of copies present in the sample of each target sequence compared to a standard reference sequence (Rp49). Samples with zero copies present (zero prevalence) were not included in quantification analyses. Pathogens assessed include: Acute Bee Paralysis Virus (ABPV), Chronic Bee Paralysis Virus (CBPV), Deformed Wing Viruses A and B (DWV-A, DWV-B), Israeli Acute Paralysis Virus (IAPV), Lake Sinai Virus (LSV), and *Nosema ceranae* (NC). There was no difference in pathogen expression between treatment groups: ABPV *F*_1_ = 0.51, *p* = 0.49; CBPV *F*_1_ = 2.79, *p* = 0.06; DWV-A *F*_1_ = 0.35, *p* = 0.56; DWV-B *F*_1_ = 0.64, *p* = 0.42; IAPV *F*_1_ = 0.50, *p* = 0.50; LSV *F*_1_ = 0.18, *p* = 0.67; NC *F*_1_ = 0.08, *p* = 0.78.

## Abbreviations

The following abbreviations are used in this manuscript:

PRF-1	Pollen-Replacing Feed 1
