# Supplementary material for: Enhanced Honey Bee Colony Strength and Economic Returns from Fall and Winter Feeding with a Complete Pollen-Replacing Feed
Source: Insects. 2026 Feb 26;17(3):243. doi: 10.3390/insects17030243 (PMC13026832; doi:10.3390/insects17030243)

Supplementary Material

Figure S1. Mean Varroa loads (mites per 100 bees) per colony in PRF-1 (n = 120) and Commercial Standard-fed (n = 120) colonies at the start of the study in fall and in January. There was no difference in Varroa load between treatment groups ($F_1 = 0.1397$ $p = 0.71$).

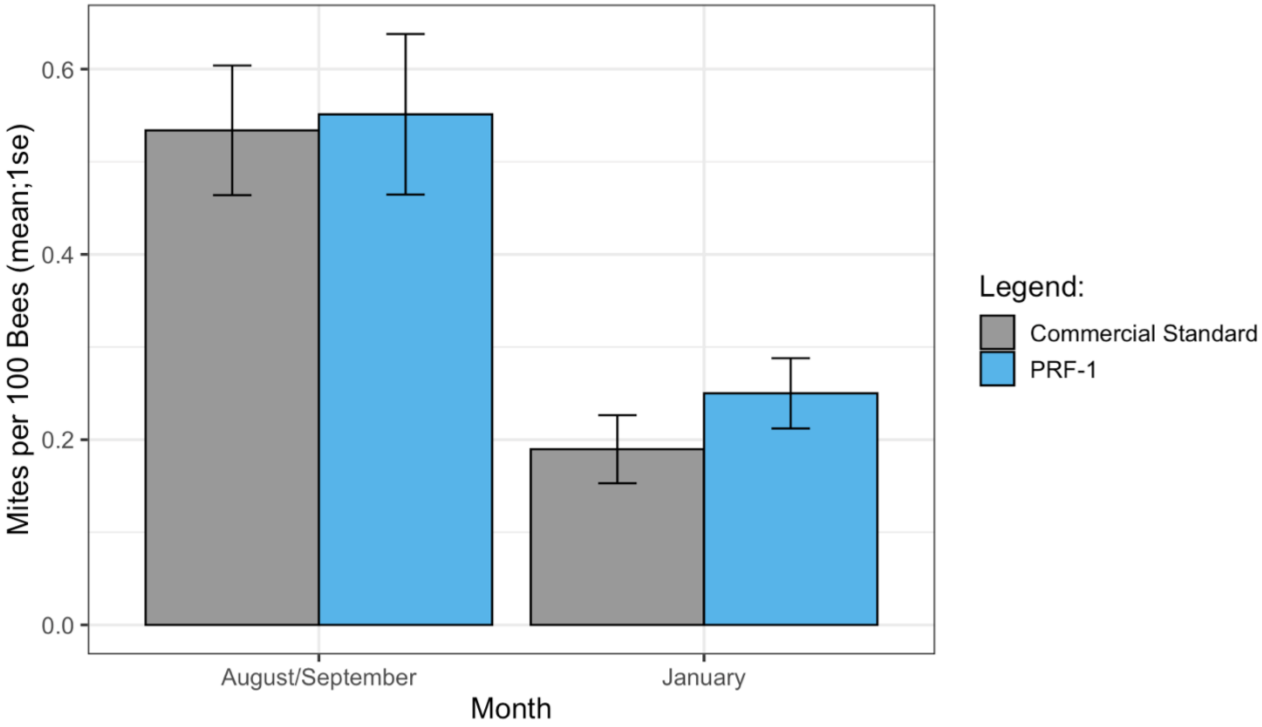

Figure S2. Pathogen prevalence (proportion of samples with each pathogen detected) in PRF-1 ($n = 60$) and Commercial Standard-fed ($n = 60$) colonies at the start of the study in fall and in January. Pathogens assessed include: Acute Bee Paralysis Virus (ABPV), Chronic Bee Paralysis Virus (CBPV), Deformed Wing Viruses A and B (DWV-A, DWV-B), Israeli Acute Paralysis Virus (IAPV), Kashmir Bee Virus (KBV), Lake Sinai Virus (LSV), and *Nosema ceranae* (NC). There were no significant differences between treatment groups for any pathogen: ABPV $F_1 = 2.91$, $p = 0.09$; CBPV $F_1 = 0.01$, $p = 0.94$; DWV-A $F_1 = 0.15$, $p = 0.70$; DWV-B $F_1 = 0.00$, $p = 1.00$; IAPV $F_1 = 0.001$, $p = 0.97$; KBV $F_1 = 0.00$, $p = 1.00$; LSV $F_1 = 3.74$, $p = 0.06$; NC $F_1 = 0.24$, $p = 0.62$.

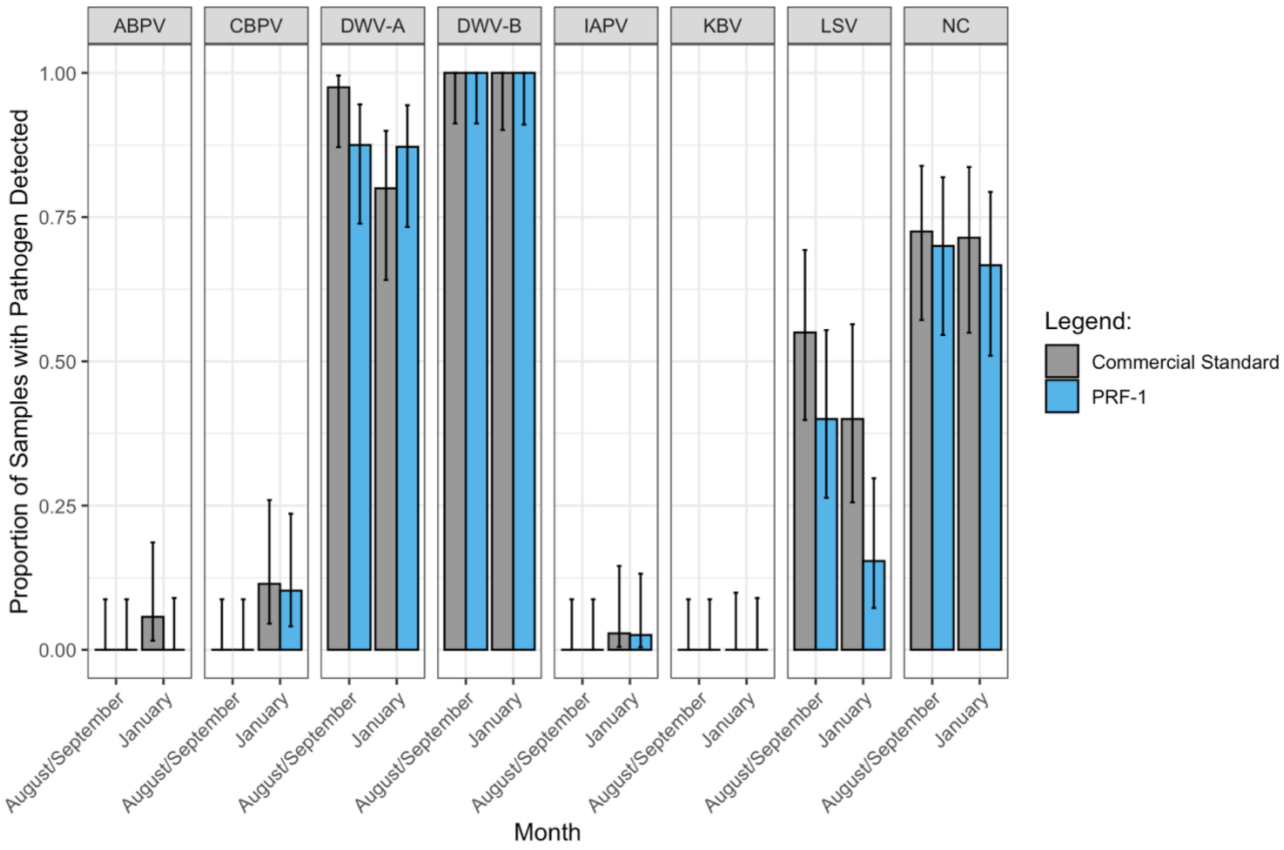

Figure S3. Fold difference in quantified pathogen expression between PRF-1 (*n* = 60) and Commercial Standard-fed (*n* = 60) colonies at the start of the study in fall and in January. Pathogen expression is presented as the fold difference between the number of copies present in the sample of each target sequence compared to a standard reference sequence (Rp49). Samples with zero copies present (zero prevalence) were not included in quantification analyses. Pathogens assessed include: Acute Bee Paralysis Virus (ABPV), Chronic Bee Paralysis Virus (CBPV), Deformed Wing Viruses A and B (DWV-A, DWV-B), Israeli Acute Paralysis Virus (IAPV), Lake Sinai Virus (LSV), and *Nosema ceranae* (NC). There was no difference in pathogen expression between treatment groups: ABPV $F_1 = 0.51$, $p = 0.49$; CBPV $F_1 = 2.79$, $p = 0.06$; DWV-A $F_1 = 0.35$, $p = 0.56$; DWV-B $F_1 = 0.64$, $p = 0.42$; IAPV $F_1 = 0.50$, $p = 0.50$; LSV $F_1 = 0.18$, $p = 0.67$; NC $F_1 = 0.08$, $p = 0.78$.

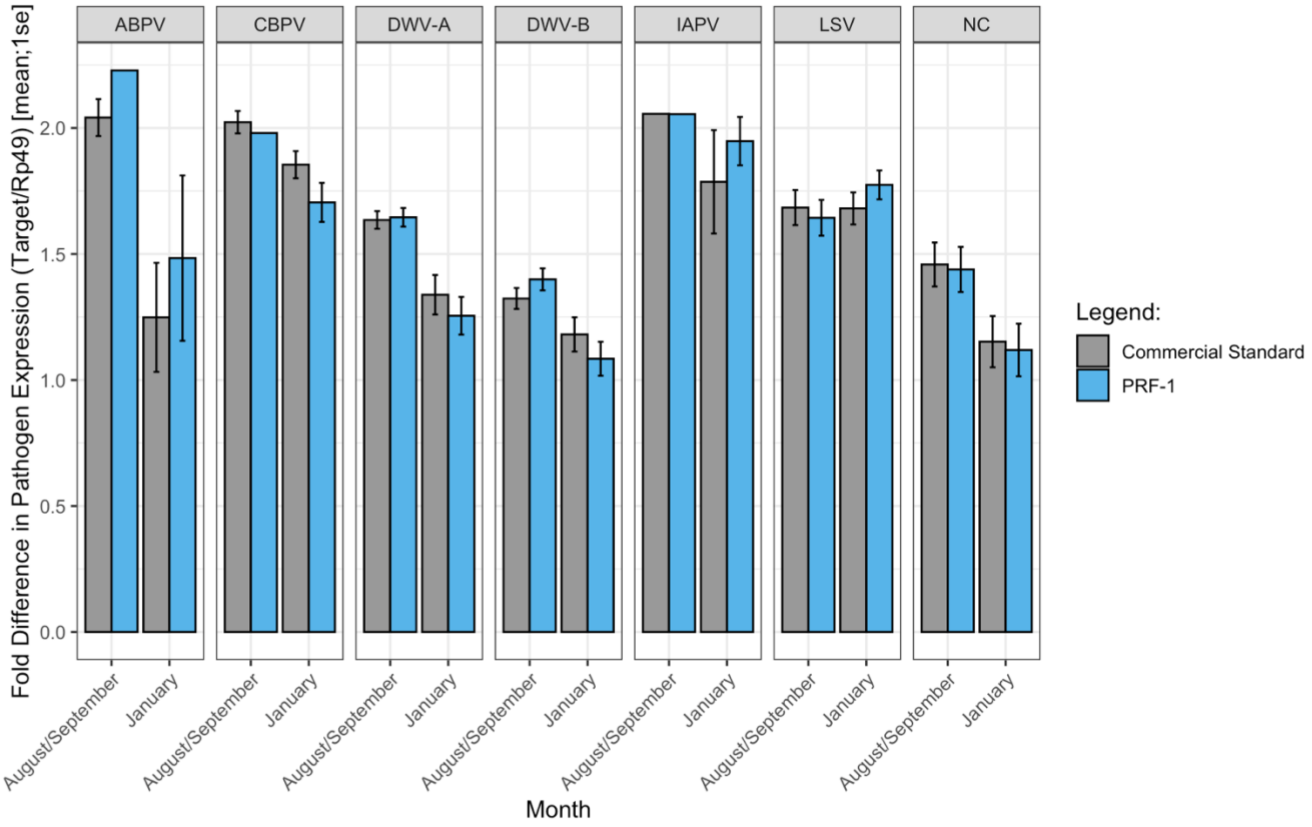

Supplement: Supplementary file 1 [file insects-17-00243-s001.zip › insects-4098996-supplementary.pdf]
